# HIV-DRIVES: HIV drug resistance identification, variant evaluation, and surveillance pipeline

**DOI:** 10.1099/acmi.0.000815.v3

**Published:** 2024-07-17

**Authors:** Stephen Kanyerezi, Ivan Sserwadda, Aloysious Ssemaganda, Julius Seruyange, Alisen Ayitewala, Hellen Rosette Oundo, Wilson Tenywa, Brian A. Kagurusi, Godwin Tusabe, Stacy Were, Isaac Ssewanyana, Susan Nabadda, Maria Magdalene Namaganda, Gerald Mboowa

**Affiliations:** 1 Department of Immunology and Molecular Biology, School of Biomedical Sciences, College of Health Sciences, Makerere University, P.O Box 7072 Kampala, Uganda; 2 The African Center of Excellence in Bioinformatics and Data-Intensive Sciences, the Infectious Diseases Institute, College of Health Sciences, Makerere University, P.O Box 22418 Kampala, Uganda; 3 National Health Laboratories and Diagnostics Services, Central Public Health Laboratories, Ministry of Health, P.O Box 7272 Kampala, Uganda; 4 Amsterdam Institute for Global Health and Development (AIGHD), Department of Global Health, Academic Medical Center, Amsterdam, Netherlands; 5 Department of Medical Microbiology and Infectious Diseases, University of Manitoba, Winnipeg, MB, Canada; 6 Africa Centres for Disease Control and Prevention, African Union Commission, Roosevelt Street, P.O. Box 3243, W21 K19 Addis Ababa, Ethiopia

**Keywords:** bioinformatics, HIV drug resistance, mutations, monitoring, next-generation sequencing

## Abstract

The global prevalence of resistance to antiviral drugs combined with antiretroviral therapy (cART) emphasizes the need for continuous monitoring to better understand the dynamics of drug-resistant mutations to guide treatment optimization and patient management as well as check the spread of resistant viral strains. We have recently integrated next-generation sequencing (NGS) into routine HIV drug resistance (HIVDR) monitoring, with key challenges in the bioinformatic analysis and interpretation of the complex data generated, while ensuring data security and privacy for patient information. To address these challenges, here we present HIV-DRIVES (HIV Drug Resistance Identification, Variant Evaluation, and Surveillance), an NGS-HIVDR bioinformatics pipeline that has been developed and validated using Illumina short reads, FASTA, and Sanger *ab1*.seq files.

**Impact Statement**

Approximately 39.0 million people were living with HIV in 2022, with at least 89 % accessing antiretroviral therapy (ART). However, in the same year, 1.3 million people were newly infected with HIV globally. The emergence of HIV drug resistance (HIVDR) has been identified as a major challenge to treatment success and has compromised the effectiveness of ART in reducing HIV incidence and HIV-associated morbidity and mortality. HIV variants within an infected individual are not genetically identical but form pools of highly diversified viruses. Traditional HIVDR testing relies on sequencing of relevant HIV genes using Sanger sequencing to detect known HIVDR mutations, but this method is unable to quantitatively identify mutations at frequencies below 20 %, yet these mutations have been implicated in HIVDR. Sanger technology is steadily being replaced with next-generation sequencing (NGS) as a new standard for HIVDR testing during virological failure, before ART initiation, or during ART regimen switch. The COVID-19 pandemic boosted NGS testing in many public health laboratories, especially in low- and middle-income countries. These laboratories are now poised to perform NGS-based HIVDR testing, largely due to its massive parallel data throughput and scalability. However, the volume and complexity of data generated require more user-friendly automated bioinformatics analysis pipelines. Recognizing this, we introduce the HIV-DRIVES (HIV Drug Resistance Identification, Variant Evaluation, and Surveillance) bioinformatics pipeline. This high-level analytical pipeline has been purposefully designed to overcome the limitations inherent in traditional HIVDR profiling methods. HIV-DRIVES (https://github.com/MicroBioGenoHub/HIV-DRIVES) is an open-source, user-friendly, command-line, and scalable pipeline that generates a PDF report that can easily be shared and interpreted by clinicians.

## Data Summary

The source code and operation manual for HIV-DRIVES are available from GitHub under GNU GPL v3; (https://github.com/MicroBioGenoHub/HIV-DRIVES). The authors confirm that all supporting data, code, and protocols have been provided within the article. The genomic raw reads files from this study are publicly available at the Sequence Read Archive (SRA) of the National Center for Biotechnology Information (NCBI) under the study BioProject ID: PRJNA1024060. The respective accession IDs are in File S4 (available in the online Supplementary Material).

Questions and issues can be sent to kanyerezi30@gmail.com or ivangunz23@gmail.com. The future directions of HIV-DRIVES include implementation within Singularity, Docker and Nextflow platform containers as well as the integration of further enhancements in terms of scalability and usability.

## Introduction

The introduction of lifelong human immunodeficiency virus (HIV) combination antiretroviral therapy (cART) is now saving millions of lives globally. However, the rise of drug-resistant strains of HIV presents a formidable challenge in effectively managing HIV infections [1]. Precise identification and evaluation of HIV drug resistance (HIVDR) mutations are essential for guiding appropriate treatment decisions and devising effective therapeutic strategies [2]. Consequently, the demand for advanced bioinformatics pipelines that seamlessly integrate drug resistance identification, variant evaluation, and surveillance has become increasingly evident.

Traditionally, HIVDR profiling relied on tools such as the HIVdb program, a web-based program hosted by Stanford University, California, USA, along with RECall and HyDRA, following the sequencing process [3,6]. While these tools have been invaluable in shedding light on drug resistance mutations, they exhibit limitations concerning data protection, transmission, and automation of the analysis. With web-based tools, concerns arise over the potential compromise of patient privacy due to the transmission of patient data over networks beyond countries' borders. In contrast, certain command-line tools conduct analyses in fragments, lacking the ability to offer a comprehensive end-to-end analysis and provide easily interpretable portable clinically actionable results. Additionally, these tools may not support the analysis of genomic data from various sequencing platforms.

To address these challenges, we introduce the HIV-DRIVES (HIV Drug Resistance Identification, Variant Evaluation, and Surveillance) bioinformatics pipeline. This high-level analytical pipeline has been purposefully designed to overcome the limitations inherent in traditional HIV drug resistance profiling methods. HIV-DRIVES offers a seamless and efficient approach for the detection, evaluation, and monitoring of HIV drug resistance mutations, harnessing the capabilities of advanced sequencing data and computational techniques. The development of HIV-DRIVES has been motivated by the goal of enhancing both patient care and public health, capitalizing on the potential of next-generation sequencing (NGS) technologies.

## Implementation

### Data generation

The pipeline was tested on data archived and generated by the National Genomics Reference Laboratory housed at the Central Public Health Laboratories (CPHL). The National Genomics Reference Laboratory in its mandate receives samples with viral loads of >1000 copies µl^−1^ all over Uganda for HIV drug resistance profiling to guide the treatment of patients as stated in the National HIV management guidelines. For the development of HIV-DRIVES, 178 samples were randomly selected and subjected to the processes below. Among these samples, 84 were males with a mean age of 25, and a mean viral load of 220  267, and 94 were females with a mean age of 26 and a mean viral load of 89 341 (File S3). RNA was extracted using the QIAamp viral RNA extraction kit following the in-house customized protocol. The extracted RNA was reverse transcribed to cDNA, which was later amplified using the respective primer sets to generate amplicons. Quality assessment of the amplicons was performed using gel electrophoresis. Library preparation for the good-quality amplicons was performed using the Illumina DNA prep kit. To assess the quality of prepared libraries, DNA quantification, and normalization using the qubit4 Fluorometer and library size estimation using Agilent bioanalyzer were performed. The genomic libraries were loaded onto both the Illumina MiSeq and iSeq platforms for sequencing at the National Genomics Reference Laboratory.

### Pipeline architecture

HIV-DRIVES is a tool designed with three programming languages, Shell script, R, and Perl. It was compiled and tested on Ubuntu 18.0.4 LTS (Bionic Beaver), WSL Ubuntu 20.04.1 LTS (Focal Fossa), and WSL Ubuntu 18.04.1 LTS (Focal Fossa). HIV-DRIVES was put together using different packages that include trim_galore, Bowtie 2, SAMtools, Quasitools, and Sierra-local [6,10]. All these packages and their derivatives are housed in the HIV-DRIVES conda environment so that they do not interfere with already existing programs. The HIV-DRIVES help message was adapted from the rMAP help message and edited to suit the context of HIV-DRIVES [11]. The full list of the dependent packages is provided in Table 1.

**Table 1.** Packages housed in HIV-DRIVES

Package name	Version	Summary
Trim_galore	0.6.10	Quality assessment of reads
Bowtie	2.5.1	Alignment of reads
SAMtools	1.7	Filtering of host reads
Quasitools	0.7.0	Variant calling and consensus genome generation
Sierra-local	–	Prediction of HIV drug resistance
R	4.1.1	Extraction of drug resistance profiles

### Overview of the HIV-DRIVES pipeline workflow

**Fig. 1.**
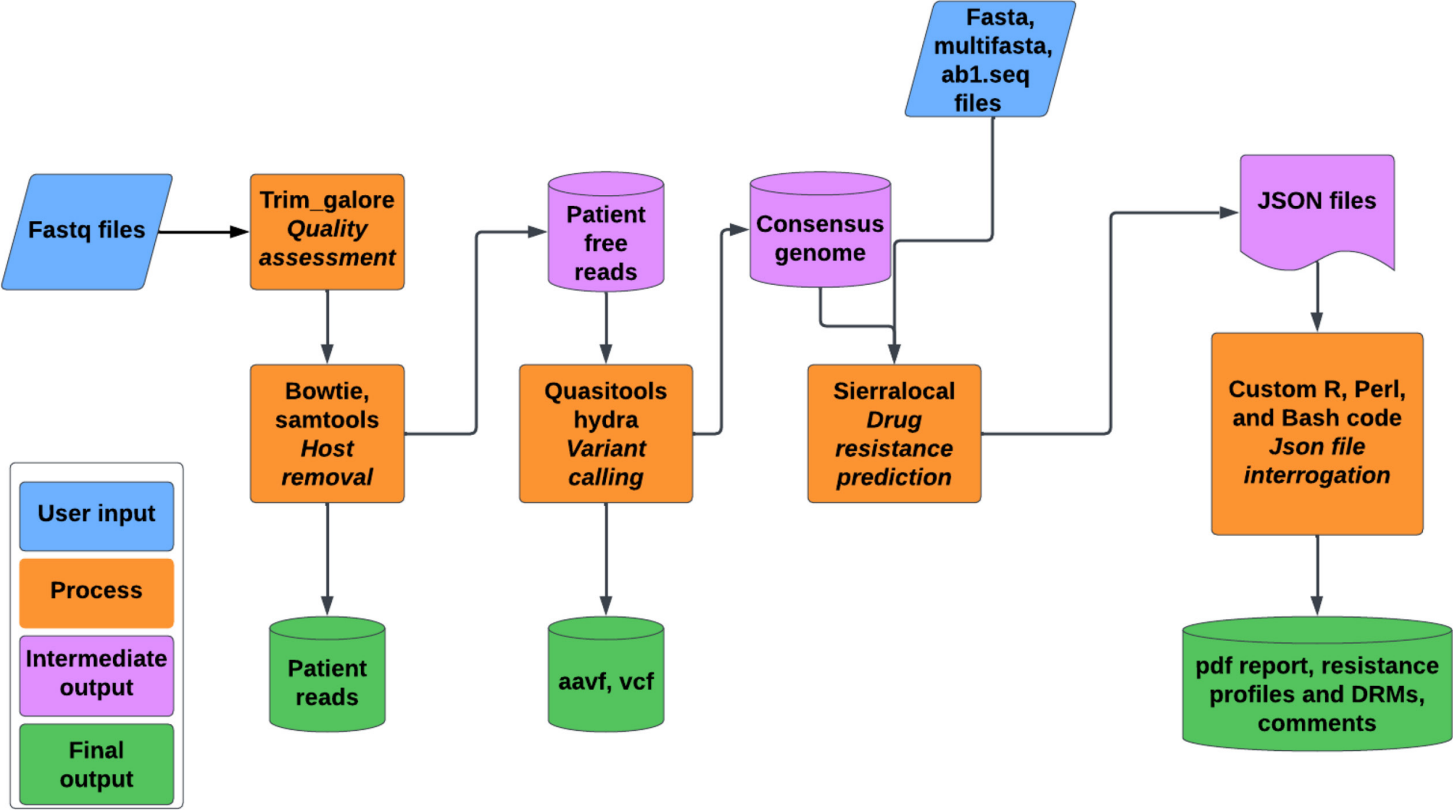
The workflow for the HIV-DRIVES pipeline.

HIV-DRIVES is designed to perform HIV drug resistance profiling and amino acid mutation detection from NGS data from Illumina platforms, Sanger sequence data in *ab1*.seq format, and FASTA files. Given input data as FASTQ files, the tool uses trim_galore to filter out reads at a threshold phred score of *q*28. The remaining reads are aligned to the host genome (GRCh38) using Bowtie 2 to separate host and viral reads using SAMtools. The resultant viral reads are subjected to HyDRA from Quasitools to generate a consensus genome and detect amino acid variants. The resultant consensus genome is subjected to Sierra-local to predict the drug resistance within the genome. The output json file from Sierra-local and amino acid mutations from HyDRA are interrogated using customized R, Perl, and Bash code to match the drug resistance profiles and their corresponding mutations plus comments, which are finally output in a PDF file. The PDF file consists of three tables. The first table consists of the drug classes, their corresponding drugs, resistance profiles (which are color-coded), and the drug resistance mutations that contribute to the resistance profiles. The second table gives the full names of the abbreviated drug names, and the third table gives the comments about the drugs. For Sanger and FASTA files, the tool uses Sierra-local to determine the drug resistance within the genome (Fig. 1).

HIV-DRIVES can be run in three different modes, namely, all, varcall, and resistance. If someone has FASTQ files and would like to obtain resistance profiles at the end of the run, then they will need to turn on all modes and the corresponding options. If someone has FASTQ files and they only want to obtain the amino acid mutations and a consensus genome FASTA file, they will need to turn on the varcall mode and the corresponding options. If someone has a consensus genome FASTA file and would like to obtain resistance profiles at the end of the run, then they will need to turn on the resistance mode and the corresponding options. The resistance mode works for those with a consensus file in both FASTA and multifasta format, and those with a Sanger output file in the format of *ab1*.seq. For all scenarios that require resistance profiling, the pipeline starts by updating the HIVDB resistance algorithm. The procedure for running all these modes is described in the core pipeline features section.

### Core pipeline features

The core parameters of HIV-DRIVES are dependent on which mode the user is interested in running, which is dependent on one’s needs, but the output directory is a mandatory parameter for all the modes. Here, we describe how to run the pipeline with different modes and the corresponding needs.

### All

The all mode is to be run when someone has FASTQ files that are either paired or single-ended and they want to obtain resistance profiles at the end.

For paired reads, below is how to run the tool:

HIV-DRIVES -o<output directory to be created > -f<path to the forward read > -r<path to the reverse read > --all true

For single-ended reads, below is how to run the tool:

HIV-DRIVES -o<output directory to be created > --single-end true --se < path to the single-ended read > --all true

### Varcall

The varcall mode is to be run when someone has FASTQ files that are either paired or single-ended and they only want amino acid mutations and consensus genome files generated at the end.

For paired reads, below is how to run the tool:

HIV-DRIVES -o<output directory to be created > -f<path to the forward read > -r<path to the reverse read > --varcall true

For single-ended reads, below is how to run the tool:

HIV-DRIVES -o<output directory to be created > --single-end true --se < path to the single-ended read > --varcall true

### Resistance

The resistance mode is run when someone has either the Sanger *ab1*.seq file format or a FASTA file. It also supports a multifasta file. If someone has a Sanger file, below is how to run the tool:

HIV-DRIVES -o<output directory to be created > --resistance true --sanger < path to the *ab1*.seq file >

If someone has a FASTA file, below is how to run the tool:

HIV-DRIVES -o<output directory to be created > --resistance true --consensus < path to the fasta file >

For all the modes, the resultant PDF files are output in the results directory under the output directory. The results directory also contains aavf, vcf, resistance profile in csv, drug resistance mutations corresponding to the drug classes, and the comments of mutations in csv files. For the all and varcall modes, the output directory has patient_free_reads and patient_reads directories in which the viral reads and patient reads are found, respectively. In cases of data sharing, the owner of the data can share the viral reads with confidence that patient genomic data are not shared.

### Testing and validation

**Fig. 2.**
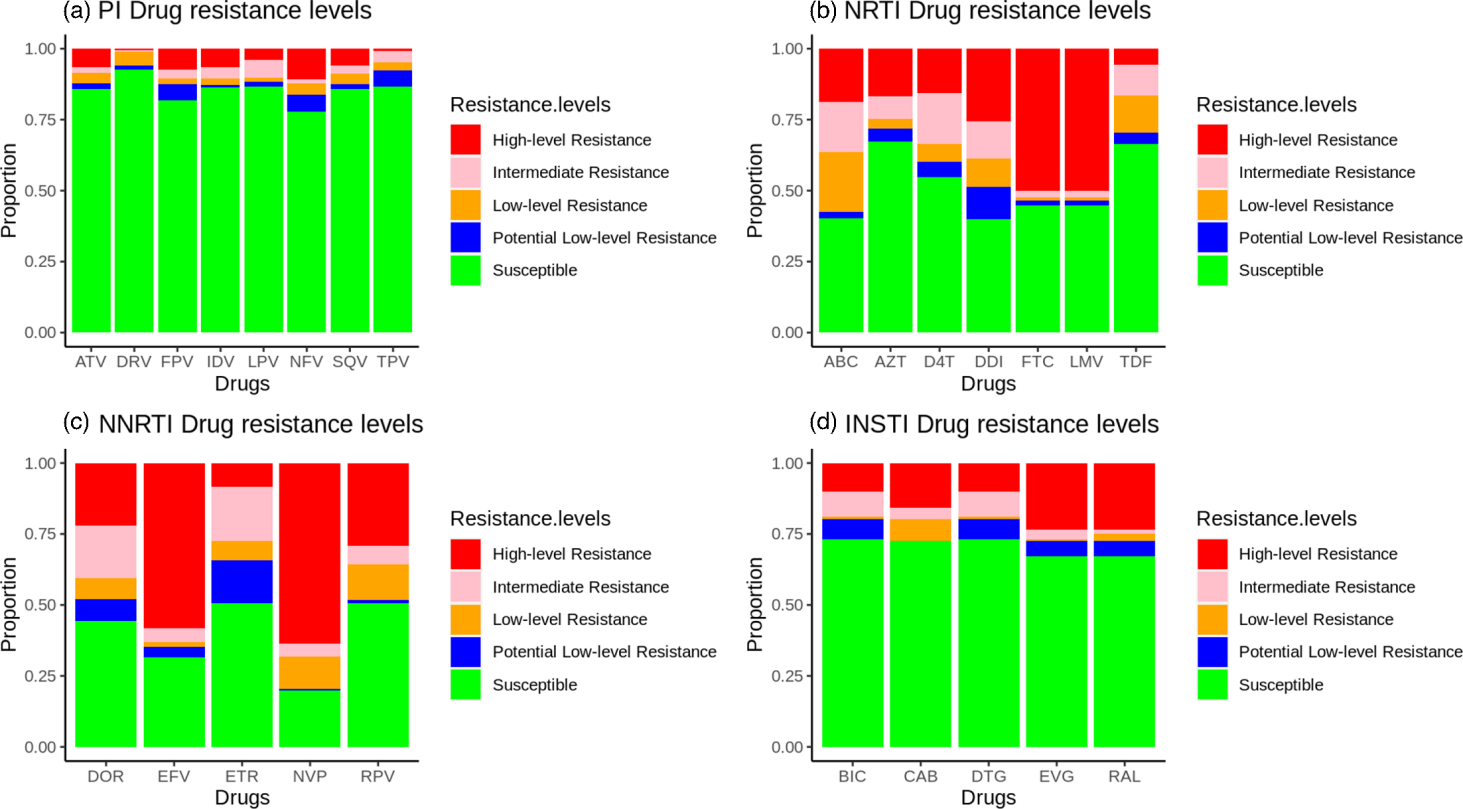
Drug resistance profiles by drug class.

**Fig. 3.**
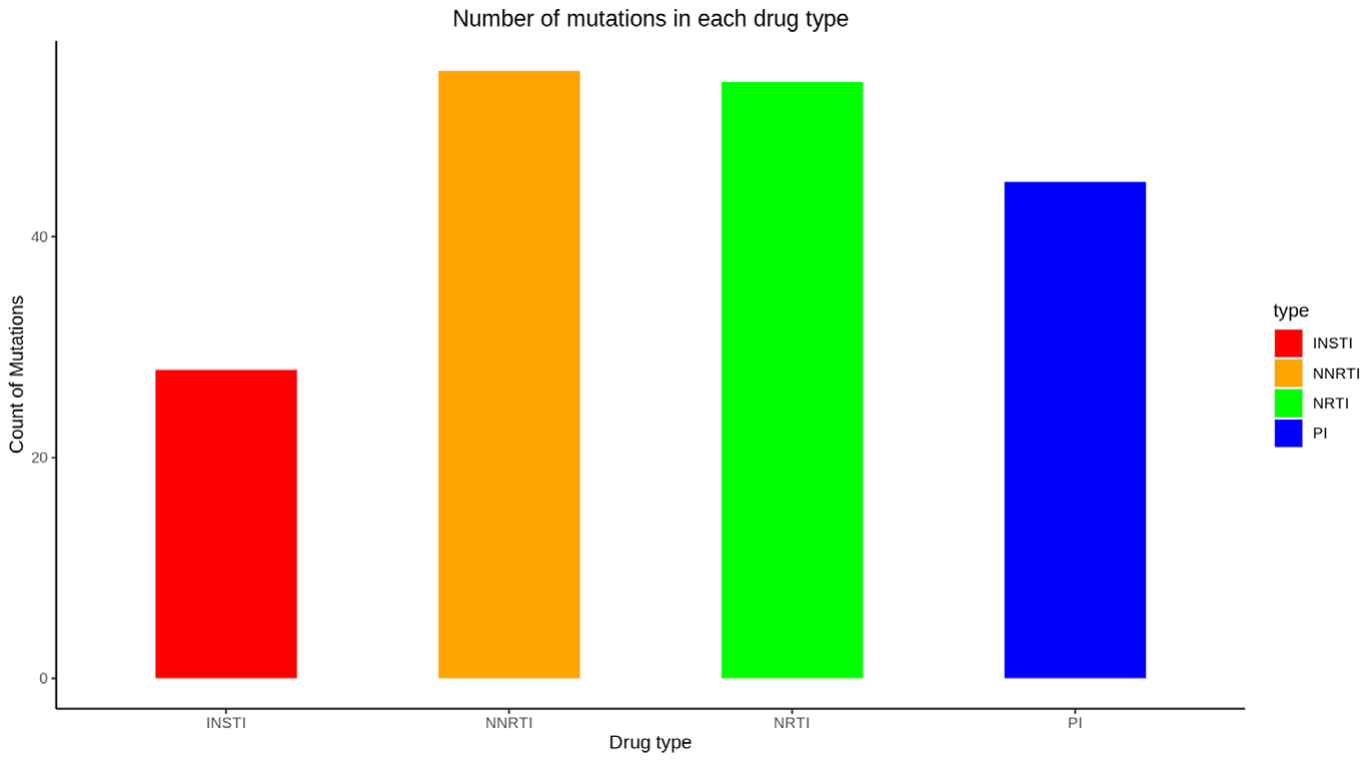
Number of mutations for each drug class.

The pipeline was tested on samples sequenced on the MiSeq and iSeq sequencing platforms at the National Genomics Reference Laboratory housed at the Uganda Central Public Health Laboratories and Illumina and Sanger samples from https://f1000research.com/articles/11-901, as well as all protease and integrase example sequences from the Stanford University HIV drug resistance database (https://hivdb.stanford.edu/hivdb/by-sequences/) [3,4, 12]. HIV-DRIVES was benchmarked with the classical Stanford University HIVdb program (https://hivdb.stanford.edu/hivdb/by-sequences/) with the resistance profiles and drug-resistant mutations that contribute to the resistance as the metrics of comparison [3,4]. For each sample, we obtained drug resistance profiles for antiretroviral drugs of the following drug classes: protease inhibitors (PIs), non-nucleoside reverse transcriptase inhibitors (NNRTIs), nucleoside reverse transcriptase inhibitors (NRTIs), and integrase strand transfer inhibitors (INSTIs) (File S1) with the drug resistance mutations contributing to the resistances (File S2). Additionally, the corresponding comments to the mutations were extracted. For all the metrics, there was 100 % concordance with the Stanford University HIVdb program. Fig. 2 provides a summary of the resistance profiles and Fig. 3 gives the number of mutations in each drug type.

For PIs and INSTIs, the samples were more susceptible to the drugs tested, as seen in Fig. 2. For NRTIs and NNRTIs, the samples were more resistant to the drugs tested. From Fig. 3, NNRTIs had the highest number of drug-resistant mutations, followed by NRTIs, PIs, and finally INSTIs. E138K, M184V, K103N, and V82A were the most abundant mutations among INSTIs, NRTIs, NNRTIs, and PIs, respectively (File S2). The validated reports for the HIV-DRIVES compared with Stanford can be found at https://github.com/MicroBioGenoHub/HIV-DRIVES-validation-reports.

To further evaluate the speed of the program, we noted the per-sample analysis time when it was run on the different platforms, as seen in Table 2.

**Table 2.** HIV-DRIVES wall clock runtimes across computer operating system platforms

Platform	RAM	CPUs	Per-sample execution time (min)
Ubuntu 18.0.4 LTS (Bionic Beaver)	16 GB	8	~5
WSL Ubuntu 20.04.1 LTS (Focal Fossa)	16 GB	8	~6
WSL Ubuntu 18.04.1 LTS (Focal Fossa)	16 GB	8	~6

## Conclusion

In summary, the HIV-DRIVES (HIV Drug Resistance Identification, Variant Evaluation, and Surveillance) bioinformatics pipeline stands as a potent and pioneering instrument within the realm of HIV drug resistance surveillance and treatment. This pipeline effectively harnesses state-of-the-art sequencing data and computational methodologies to identify, assess, and track mutations associated with HIV drug resistance. Consequently, it bolsters our capacity to comprehend and counteract HIV drug resistance, ultimately contributing to the development of more efficient treatment strategies tailored to public health needs and the enhancement of patient care. The incorporation of this pipeline into the infrastructure of the Central Public Health Laboratory (CPHL) in Uganda for routine HIVDR care not only solidifies its relevance in public health but also underscores its potential for adoption in similar settings.

## supplementary material

10.1099/acmi.0.000815.v3 Uncited Supplementary Material 1.

10.1099/acmi.0.000815.v3 Uncited Supplementary Material 2.

10.1099/acmi.0.000815.v3 Uncited Supplementary Material 3.

10.1099/acmi.0.000815.v3 Uncited Supplementary Material 4.

## Abbreviations

INSTIs: integrase strand transfer inhibitors
NNRTIs: non-nucleoside reverse transcriptase inhibitors
NRTIs: nucleoside reverse transcriptase inhibitors
PIs: protease inhibitors
